# A Novel Controllable Hydrogen Sulfide-Releasing Molecule Protects Human Skin Keratinocytes Against Methylglyoxal-Induced Injury and Dysfunction

**DOI:** 10.1159/000366339

**Published:** 2014-09-29

**Authors:** Chun-tao Yang, Yu Zhao, Ming Xian, Jian-hua Li, Qi Dong, Hong-bo Bai, Ji-de Xu, Mei-fen Zhang

**Affiliations:** a Department of Physiology, Guangzhou Medical University, Guangzhou P.R. China; b Department of Chemistry, Washington State University, Pullman, W.A. USA; c School of Nursing, Sun Yat-sen University, Guangzhou, P.R. China

**Keywords:** Delayed wound healing, Diabetes mellitus, Hydrogen sulfide, Keratinocyte, Methylglyoxal

## Abstract

**Background/Aim:**

Delayed wound healing is a common skin complication of diabetes, which is associated with keratinocyte injury and dysfunction. Levels of methylglyoxal (MGO), an α-dicarbonyl compound, are elevated in diabetic skin tissue and plasma, while levels of hydrogen sulfide (H_2_S), a critical gaseous signaling molecule, are reduced. Interestingly, the gas has shown dermal protection in our previous study. To date, there is no evidence demonstrating whether MGO affects keratinocyte viability and function or H_2_S donation abolishes these effects and improves MGO-related impairment of wound healing. The current study was conducted to examine the effects of MGO on the injury and function in human skin keratinocytes and then to evaluate the protective action of a novel H_2_S-releasing molecule.

**Methods:**

An N-mercapto-based H_2_S donor (NSHD)-1 was synthesized and its ability to release H_2_S was observed in cell medium and cells, respectively. HaCaT cells, a cell line of human skin keratinocyte, were exposed to MGO to establish an *in vitro* diabetic wound healing model. NSHD-1 was added to the cells before MGO exposure and the improvement of cell function was observed in respect of cellular viability, apoptosis, oxidative stress, mitochondrial membrane potential (MMP) and behavioral function.

**Results:**

Treatment with MGO decreased cell viability, induced cellular apoptosis, increased intracellular reactive oxygen species (ROS) content and depressed MMP in HaCaT cells. The treatment also damaged cell behavioral function, characterized by decreased cellular adhesion and migration. The synthesized H_2_S-releasing molecule, NSHD-1, was able to increase H_2_S levels in both cell medium and cells. Importantly, pretreatment with NSHD-1 inhibited MGO-induced decreases in cell viability and MMP, increases in apoptosis and ROS accumulation in HaCaT cells. The pretreatment was also able to improve adhesion and migration function.

**Conclusion:**

These results demonstrate that the novel synthesized H_2_S donor is able to protect human skin keratinocytes against MGO-induced injury and behavior dysfunction. We believe that more reasonable H_2_S-releasing molecules will bring relief to patients suffering from delayed wound healing in diabetes mellitus in the future.

## Introduction

Diabetic skin ulcer is a major and increasing public health concern in many parts of the world. It usually causes substantial morbidity, impairs quality of life, and results in high treatment costs for patients [1]. In the diabetic skin ulcer, the resident cells undergo phenotypic changes, and their capacity of proliferation and movement is impaired [1–3]. Therefore, it is important to discover the mechanisms underlying the dysfunction of skin cells in therapy of diabetic skin ulcer.

Methylglyoxal (MGO) is a reactive dicarbonyl agent produced in the metabolism of glucose, fatty acid and amino acid [4]. As a potent carbonyl stress agent, MGO is able to induce nonenzymatic modification of proteins and nucleotides by cross-linking biological macromolecules resulting in the formation of advanced glycation end-products (AGEs) [4]. It has been well demonstrated that AGEs, which are closely associated with diabetic complications, aging disorder, oxidative stress and chronic inflammation, can lead to cellular injury and behavior dysfunction [5, 6]. Of note, the levels of free MGO are increased in the plasma of diabetics [7, 8]. Additionally, in the diabetic skin collagen, Maillard reaction products, including MGO, are severely accumulated [9]. Added MGO has been shown to induce lots of deleterious effects in cellular systems, such as oxidative stress [10], defects in cell adhesion [6] and endothelial dysfunction [11]. We therefore speculated that MGO-induced skin cell injury and dysfunction may contribute to the delayed wound healing observed in diabetes. As such the inhibition of its effects may be a critical therapeutic target for diabetic wound.

Hydrogen sulfide (H_2_S) is a colorless, water soluble gas, with a characteristic smell of rotten eggs. Akin to nitric oxide (NO) and carbon monoxide (CO), H_2_S has traditionally been considered as a highly toxic gas and environmental hazard. However, studies have revealed that these “toxic” gases are produced enzymatically in mammals under normal conditions and exert a number of important physiological roles [12, 13]. Reportedly, in the plasma of diabetics, the levels of H_2_S were markedly reduced [14]. In addition, our previous study has shown that H_2_S was able to protect human skin keratinocytes against hypoxia-induced injury and inflammation [15]. Up to date, it is still unclear if the reduced H_2_S levels are responsible for the impaired wound healing in diabetics and if exogenous H_2_S is able to mitigate diabetic skin injury. Recently, our group has developed a series of controllable H_2_S donors, including N-mercapto-based H_2_S donors (NSHDs) [16] and perthiol-based H_2_S donors (SSHDs) [17]. These donors have exhibited promising protective effects against myocardial ischemia-reperfusion injury [17]. However, their effects in other tissues are still unknown.

In the current study, human skin keratinocytes (HaCaT cells) were exposed to MGO to set up an *in vitro* diabetic skin wound model, and then the effects of NSHD-1 were investigated by preconditioning of the cells before exposure to MGO. Our results indicated that the exposure of HaCaT cells to MGO induced cellular injury and behavioral dysfunction, which were attenuated by pretreatment with NSHD-1.

## Materials and Methods

### Materials

MGO and Hoechst 33258 were bought from Sigma-Aldrich Co. (st. Louis, MO, US). Cell counting kit-8 (CCK-8), 2′,7′-dichlorofluorescein-diacetate (DCFH-DA) and rhodamine123 (Rh123) were purchased from Dojindo Laboratory (Kyushu, Japan). DMEM medium and fetal bovine serum (FBS) were supplied by Gibico BRL (Ground Island, NY, US).

### Synthesis and analysis of NSHD-1

**Fig. 1:**
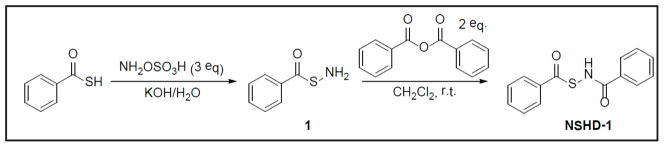
Synthesis of NSHD-1

**Fig. 2:**
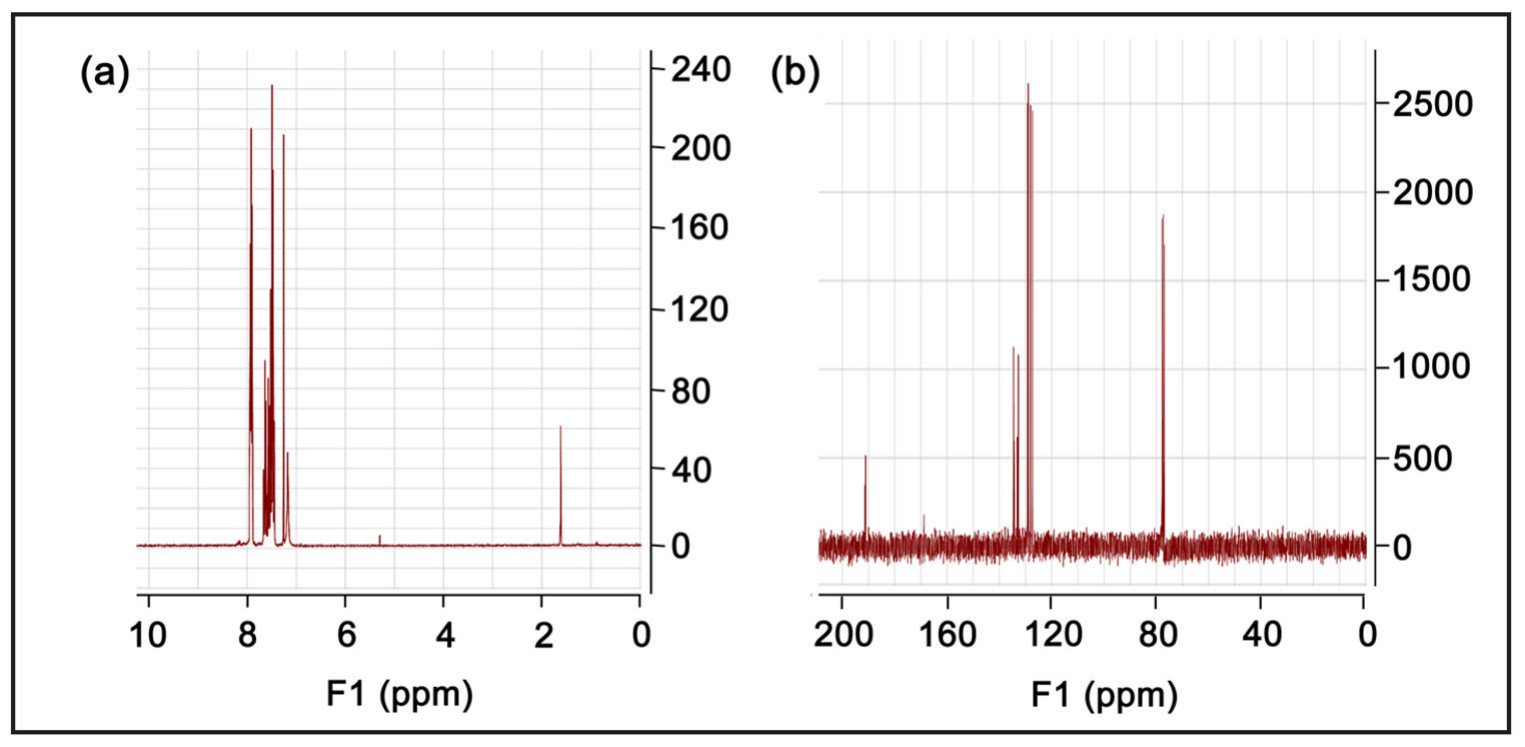
Spectra of NSHD-1. (a) ^1^H NMR (300 MHz, CDCl_3_) spectrum, (b) ^13^C NMR (75 MHz, CDCl_3_) spectrum

NSHD-1 was synthesized from the corresponding thiobenzoic acid (Fig. 1). Briefly, to a stirred solution of KOH (280 mg, 5 mmol) in water (15 mL) was added thiobenzoic acid (138 mg, 1 mmol) and hydroxylamine-*O*-sulfonic acid (339 mg, 3 mmol). The solution was stirred for 30 min at room temperature and then extracted with CH_2_Cl_2_ (X3). The solvent was removed under vacuum to afford *S*-acylthiohydroxylamine (1) as white solid. Re-dissolve 1 in CH_2_Cl_2_, followed by the addition of benzoic anhydride (452 mg, 2 mmol). The resultant solution was allowed to stir overnight at room temperature. The final product, NSHD-1, was purified by recrystallization in hexane and analyzed by ^1^H NMR and ^13^C NMR spectra (Fig. 2). m.p. 138–140 °C; ^1^H NMR (300 MHz, CDCl_3_) δ 7.92 (m, 4H), 7.60 (m, 1H), 7.53 (m, 1H), 7.39 (m, 4H), 7.17 (s, 1H); ^13^C NMR (75 MHz, CDCl_3_) δ 190.0, 159.1, 134.6, 134.4, 133.3, 132.8, 129.3, 129.0, 128.0, 127.3; IR (thin film) cm^−1^ 3265, 3062, 1696, 1659, 1451, 1419, 1257, 1207; HRMS m/z 258.0598 [M+H]^+^; calcd for C_14_H_12_NO_2_S 258.0589; overall yield: 64% (2 steps).

### Mechanisms of H_2_S generation from NSHD-1

**Fig. 3:**
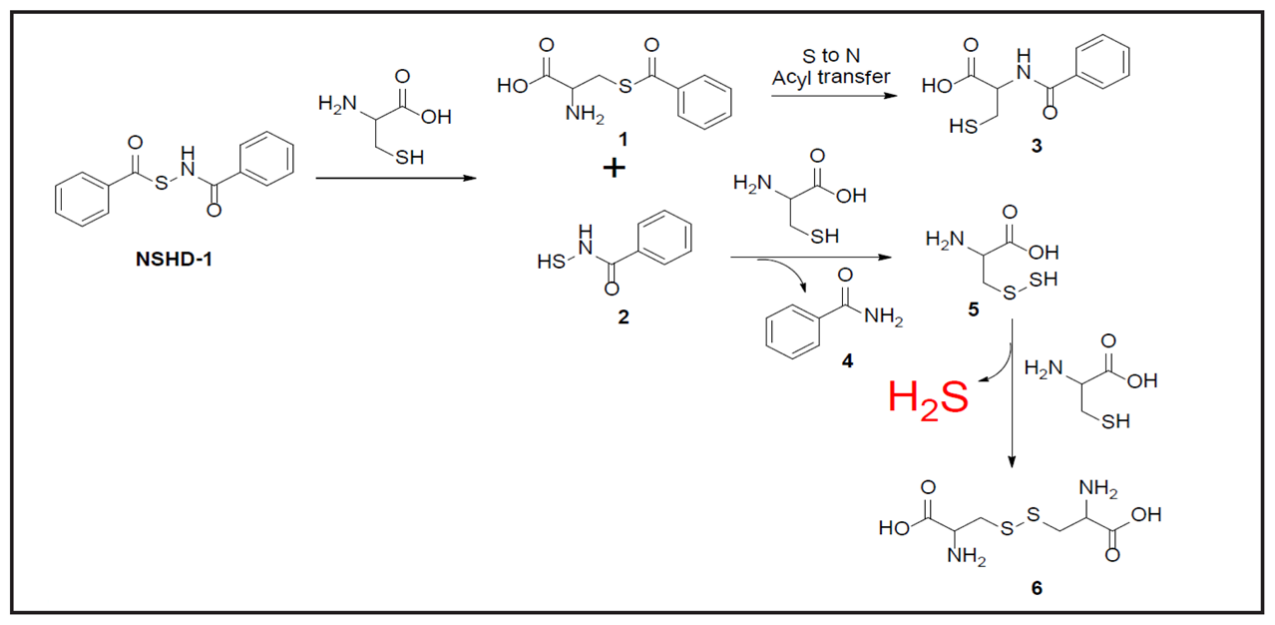
L-cysteine-activated H_2_S release

NSHD-1 has been reported to be activated by cysteine to release H_2_S [16]. Maximal quantity of 24 μM of H_2_S was detected from 40 μM of NSHD-1 in the presence of cysteine (4 mM) in aqueous buffer solution (pH 7.4). H_2_S release from NSHD-1 was also confirmed in complex biological system, such as plasma. According to the byproducts isolated, including *N*-acylcysteine, benzamide and cystine, H_2_S release mechanism was proposed as follows (Fig. 3): The reaction is initiated by reversible thiol exchange between NSHD-1 and cysteine ➀ to first generate the new thioester 1 and *N*-mercaptobenzamide 2. Compound 1 then undergoes fast S-to-N acyl transfer to form *N*-acylcysteine 3. Meanwhile, the reaction between 2 and excess cysteine ➁ should lead to benzamide 4 and cysteine perthiol 5. Finally, the reaction between 5 and cysteine ➂ should complete the generation of H_2_S and provide cystine 6. Therefore, 3 equivalents of cysteine are needed to trigger H_2_S generation from NSHD-1. However, sine commercial medium contains cystine instead of cysteine, exogenous cysteine must be added accordingly to activate NSHD-1 in the current cell experiments.

### Cell culture

HaCaT cells are derived from spontaneous transformation of human adult keratinocytes, which were bought from Kunming Institute of Zoology in Chinese Academy of Sciences (Kunming, P.R. Chian). The cells were maintained in DMEM medium supplemented with 10% FBS at 37°C under an atmosphere of 5% CO_2_ and 95% air. They were passaged and harvested using trypsin/EDTA. The culture medium was replaced every other day.

### Determination of cell viability

Cell viability assay was performed according to the manufacturer’s instruction of CCK-8 kit. HaCaT cells were plated in 96-well plates at a density of 10, 000 cells/well. When the cells were grown to approximately 70%~80% confluence, indicated treatments were applied. The CCK-8 solution (100 μL) at a 1:10 dilution with FBS-free DMEM medium was added to each well followed by a further 3 h incubation at 37°C. Absorbance (*A*) was measured at 450 nm with a microplate reader (Molecular Devices, US). Experiments were performed for 6 times.

### Assessment of apoptosis

Morphological changes of the nuclei, including chromosomal condensation and fragmentation in HaCaT cells, were observed by Hoechst 33258 staining followed by photofluorography. In brief, after the indicated treatments, the cells were fixed with 4% paraformaldehyde in phosphate buffered saline (PBS) for 10 min. After stained with 5 mg/L Hoechst 33258 for 10 min, the cells were visualized under AMG fluorescent microscope (Advanced Microscopy Group, US). Control cells displayed normal nuclear size and uniform fluorescence, whereas apoptotic cells exhibited condensed, fractured or distorted nuclei. Apoptotic rate was calculated by the ratio of the apoptotic cell number to total cell number through *Cell counter* of Image J software.

### Measurement of H_2_S content

H_2_S levels in cell medium and cells were determined by a H_2_S fluorescent probe (WSP-5) according to our recent report [18]. NSHD-1, or the same volume of dimethyl sulphoxide (DMSO) was added combined with L-cysteine and incubated for 1 h at 37°, and then 50 μmol/L WSP-5 and surfactant 100 μmol/L cetyltrimethylammonium bromide (CTAB) were added and incubated at 37° for 20 min in the dark. H_2_S-derived fluorescence was observed under AMG fluorescent microscope (Advanced Microscopy Group, US) before and after removal of cell medium in the plate wells.

### Observation of intracellular ROS accumulation

Intracellular ROS were measured by oxidative conversion of cell permeable DCFH-DA to fluorescent 2′,7′-dichlorfluorescein (DCF). After the indicated treatments, HaCaT cells were washed twice with PBS and incubated with 10 μmol/L DCFH-DA solution at 37°C for 20 min in the dark. Intercellular DCF fluorescence was observed under AMG fluorescent microscope (Advanced Microscopy Group, US). Mean fluorescence intensity (MFI) of DCF from 6 random fields was analyzed with Image J software.

### Measurement of mitochondrial membrane potential

Mitochondrial membrane potential (MMP) was examined by Rh123 staining assay followed by photofluorography. Rh123 is a cell-permeable cationic fluorescent dye that preferentially enters mitochondria based on the highly negative MMP. Depolarization of MMP brings about the loss of Rh123 from the mitochondria and a decrease in fluorescence intensity. After the treatments of HaCaT cells, 10 mg/L Rh123 dissolved in FBS-free medium was added and incubated for 20 min at 37°C. The fluorescent signal was visualized under AMG fluorescent microscope (Advanced Microscopy Group, US). The MFI of Rh123 of 6 random fields in each group was analyzed with Image J software.

### Cell adhesion assay

HaCaT cells were treated with 400 μM MGO for 48 h in the absence or presence of preconditioning with 100 μM NSHD-1 for 1 h. After the treatments, the cells were digested with 0.25% trypsin and centrifuged at 1,000×g for 5 min. The harvested cells were inoculated on 96-well plates, with 12 wells in each group. The CCK-8 solution at a 1:10 dilution with FBS-free DMEM medium was added to the six wells of each group to measure the total cells. The cells in the other six wells were cultured for a further 12 h. The medium was removed and the cells were washed with PBS twice to remove the unattached cells, and then the same CCK-8 solution was added as above to measure the adhesive cells. The adhesion rate (%) = the adhesive cells/the total cell × 100%.

### Cell migration assay

Cell migration was observed with an *in vitro* scratch assay performed as described previously with certain modifications [19]. Briefly, HaCaT cells were inoculated on 6-well plates and cultured up to about 70% confluence. Prior to the indicated treatments, a narrow wound-like gap in a cell monolayer was created with a pipette tip. And then the cells were treated with 400 μM MGO for 48 h in the absence or presence of preconditioning with 100 μM NSHD-1 for 1 h. The images were captured at 24 h and 48 h to observe the closure of gaps.

### Statistical analysis

All the data were expressed as mean ± SE. The significance of inter-group differences was evaluated by one-way analysis of variance (ANOVA) followed by LSD-*t* test with SPSS13.0 software. A probability of *P* < 0.05 was accepted as the level of statistical significance.

## Results

### MGO induced dermal injury in HaCaT cells

**Fig. 4:**
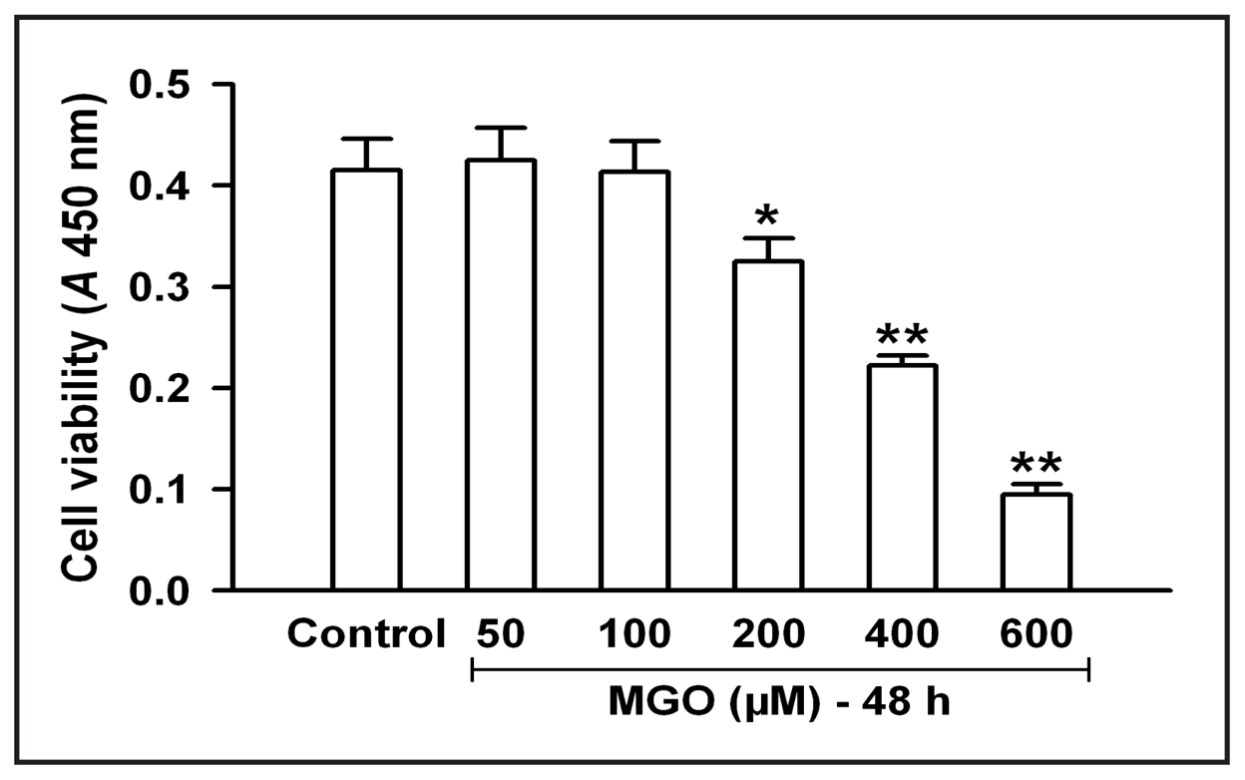
Effects of MGO on the viability of HaCaT cells The cells were exposed to indicated concentrations of MGO ranging from 50 μM to 600 μM for 48 h. Cell viability was measured by CCK-8 assay. Data were shown as the mean ± SE. n=6. ^*^P<0.05, ^**^P<0.01 compared with control group.

Since the levels of MGO were increased in diabetic plasma and skin collagen, we firstly performed experiments to test the effects of increasing concentrations of MGO on the viability in cultured human skin keratinocytes, HaCaT cells. As shown in Fig. 4, when the concentrations exceeded 200 μM, exposure to MGO induced a dose-dependent inhibition of viability with a correlation coefficient of −0.99. In addition, the result also indicated that MGO median lethal concentration in HaCaT cells was 400 μM. We therefore used this concentration of MGO to treat the cells as an *in vitro* model of diabetic wound healing.

### NSHD-1 attenuated MGO-induced injury in HaCaT cells

**Fig. 5:**
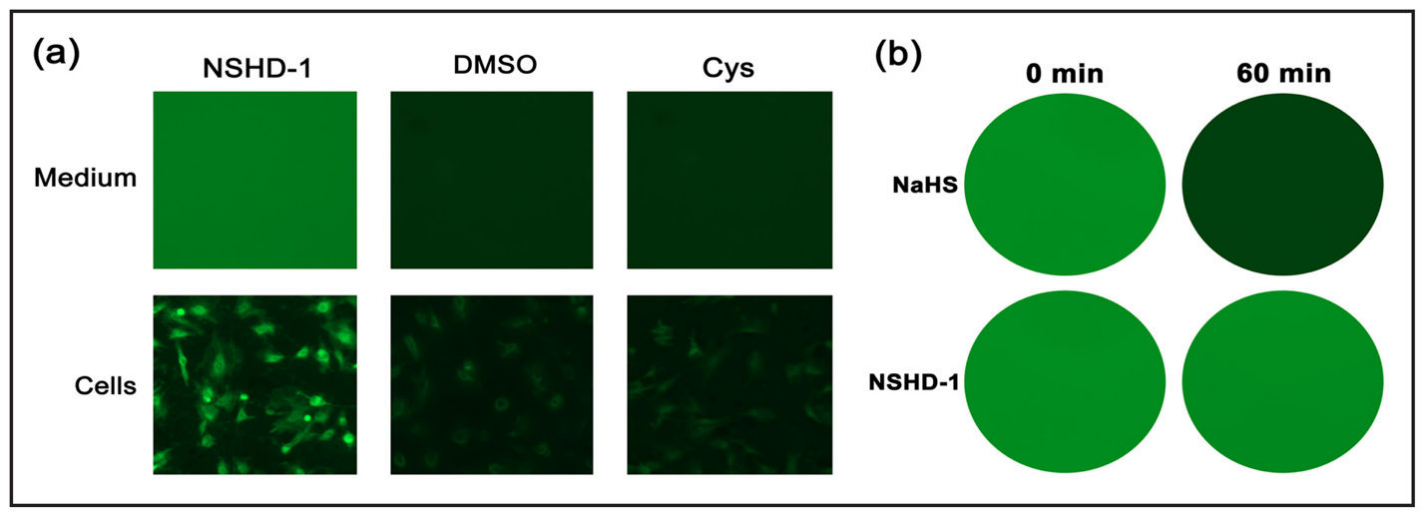
H_2_S production from donors Panel a: The cells cultured in a 12-well plate were incubated with 100 μM NSHD-1 or DMSO of the same volume combined with 300 μM L-cysteine (Cys) for 1 h, or 300 μM Cys for 1 h. Then 50 μM H_2_S fluorescent probe (WSP-5) and 100 μM CTAB were added. After incubation for 20 min, cell medium in plate wells was observed under a fluorescence microscopy and the images were taken (Top row). Following removal of the medium, intracellular H_2_S-dervied florescence was observed and the images were taken (Bottom row). Panel b: The same concentration (100 μM) of NaHS or NSHD-1 solution was added to a 12-well plate, and H_2_S-dervied fluorescence was observed at the moment of adding (0 min) or after 60 min and the images were taken.

**Fig. 6:**
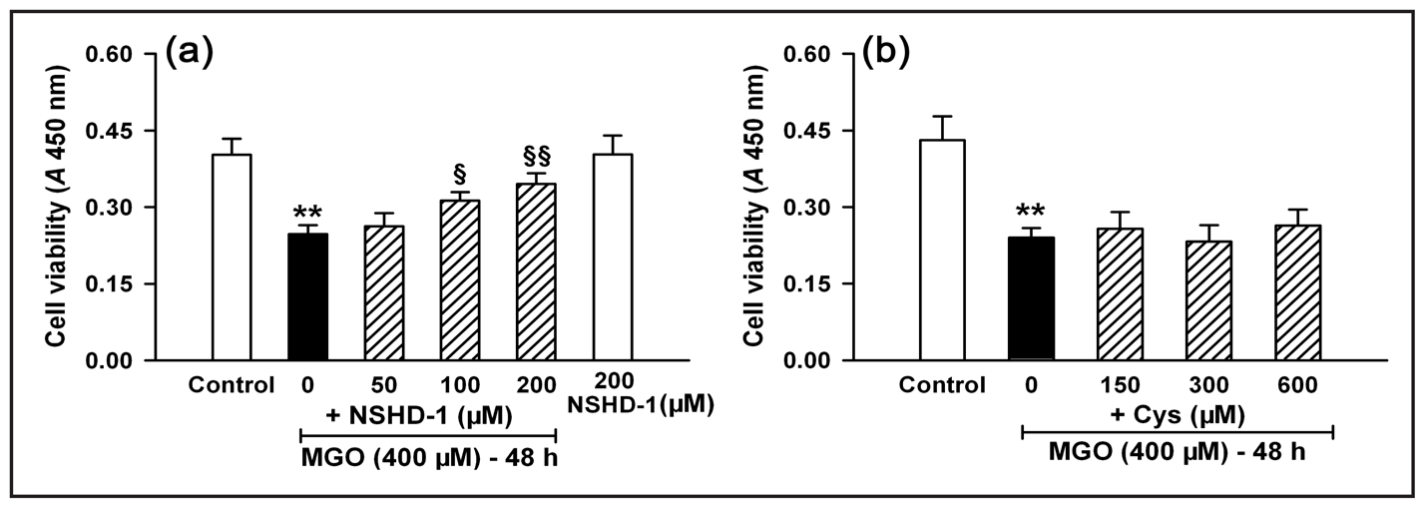
Effects of NSHD-1 on MGO-induced injury in HaCaT cells Panel a: The cells were preconditioned with various concentrations of NSHD-1 ranging from 50 μM to 200 μM combined with triple concentrations of Cys for 1 h followed by treatment with 400 μM MGO for 48 h, or with 200 μM NSHD-1 combined with 600 μM Cys for 1 h followed by a 48-h normal culture. Panel b: The cells were preconditioned with various concentrations of Cys ranging from 150 μM to 600 μM for 1 h followed by treatment with 400 μM MGO for 48 h. After the treatments, cell viability was measured by CCK-8 assay. Data were shown as the mean ± SE. n = 6. ^**^P<0.01 versus control group, ^§^*P*<0.05, ^§§^*P*<0.01 versus MGO alone group.

To examine whether NSHD-1 exerted dermal protection in MGO-treated HaCaT cells, we observed its ability of H_2_S release using a H_2_S probe WSP-5 followed by photofluorography. As presented in Fig. 5a, both in the cell medium and in the cells H_2_S-derived fluorescence were increased after the administration of 100 μM NSHD-1 for 1 h, comparing with that of DMSO or L-cysteine alone group, indicating that under the control of L-cysteine NSHD-1 has ability to release H_2_S. We next compared the stability of NSHD-1 with a traditional H_2_S donor, NaHS. As shown in Fig. 5b, NaHS-mediated H_2_S fluorescence was obviously attenuated after a 60 min incubation. However, NSHD-1 was more stable, evidenced by little change of H_2_S fluorescence during the 60 min incubation. Finally, we investigated the effects of NSHD-1 on MGO-induced cellular injury in HaCaT cells through preconditioning the cells with indicated concentrations of NSHD-1 before exposure of HaCaT cells to 400 μM MGO for 48 h. The result in Fig. 6a showed that preconditioning with NSHD-1 at 100 and 200 μM for 1 h protected HaCaT cells against MGO-induced injury, and 200 μM NSHD-1 alone didn’t alter cell viability. Since NSHD-1-mediated H_2_S release needs the activation with L-cysteine at triple concentration, we examined the effects of L-cysteine alone on MGO-induced cell injury and no positive results were found (Fig 6b). These results suggest that NSHD-1 has ability to release H_2_S and protect human skin keratinocytes against MGO-related injury.

### NSHD-1 suppressed MGO-induced apoptosis in HaCaT cells

**Fig. 7:**
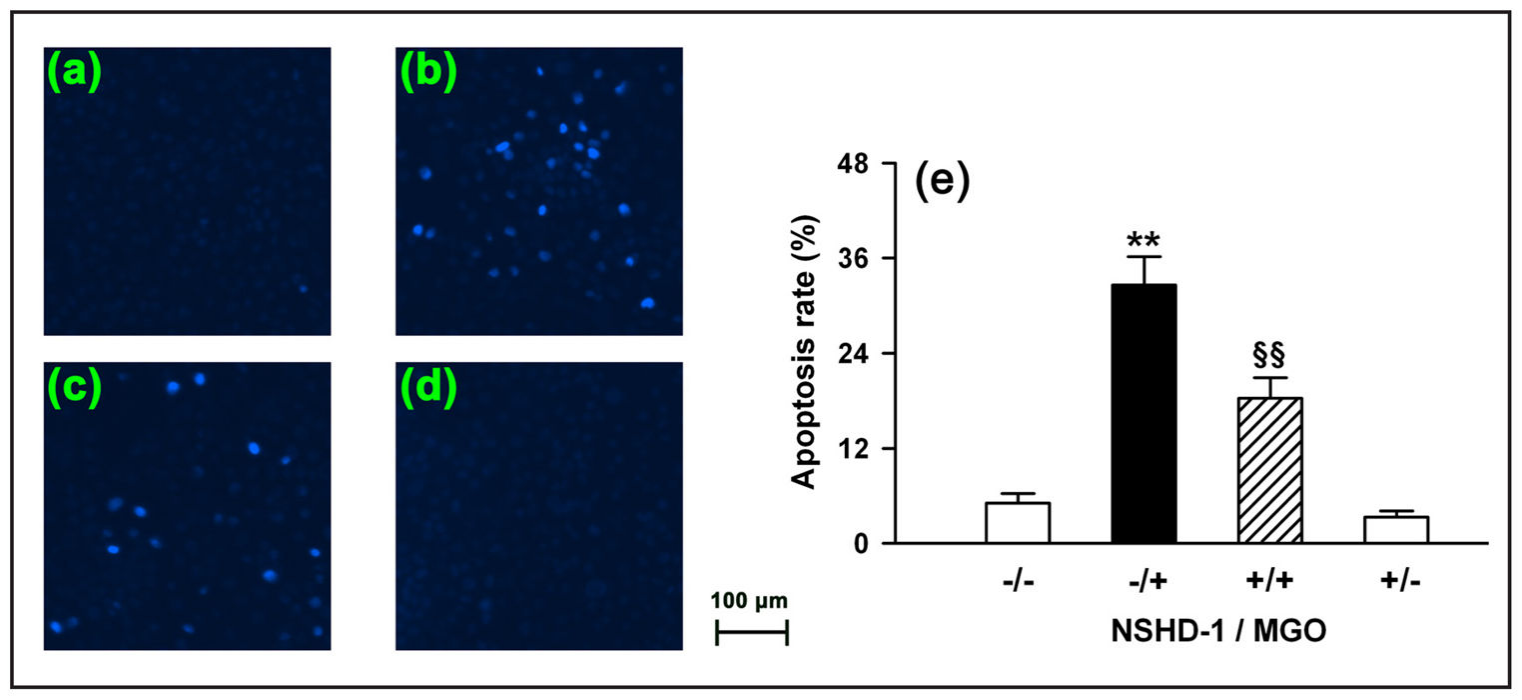
Effects of NSHD-1 on MGO-induced cellular apoptosis in HaCaT cells Hoechst 33258 nuclear staining followed by photofluorography to observe cellular apoptosis. Random Hoechst 33258 fluorescent micrographs of the (a) control, (b) treatment with 400 μM MGO for 48 h, (c) treatment with 400 μM MGO for 48 h in the presence of preconditioning with 100 μM NSHD-1 for 1 h and (d) treatment with NSHD-1 alone for 1 h followed by a 48-h culture group. During preconditioning of the cells, 300 μM Cys was contained in the cell medium of all the groups. (e) Apoptosis rate was calculated by counting normal cells and apoptotic cells from 6 random vision fields using cell counter module of Image J software. Apoptosis rate (%) = apoptosis cells/(apoptosis cells + normal cells) ×100%. Data were shown as the mean ± SE. ^**^P<0.01 versus control group, ^§§^*P*<0.01 versus MGO alone group.

Decreased cell viability may be resulted from several reasons, such as necrosis, apoptosis and autophagy. In order to reveal the mode by which MGO induced cellular injury or NSHD-1 exerted protection, experiments were performed to observe the nuclear morphological changes with Hoechst 33258 staining followed by photofluorography. As shown in Fig. 7, the nuclei of control cells (Fig. 7a) or NSHD-1 alone-treated cells (Fig. 7d) exhibited weak and uniform fluorescence, while the nuclei of cells treated with 400 μM MGO for 48 h (Fig. 7b) appeared obvious condensation and strong fluorescence, indicating cellular apoptosis. However, MGO-induced apoptosis in HaCaT cells was markedly attenuated by preconditioning with 100 μM NSHD-1 for 1 h (Fig. 7c), suggesting an anti-apoptotic action of the H_2_S donor.

### NSHD-1 impeded MGO-induced mitochondrial injury and oxidative stress in HaCaT cells

**Fig. 8:**
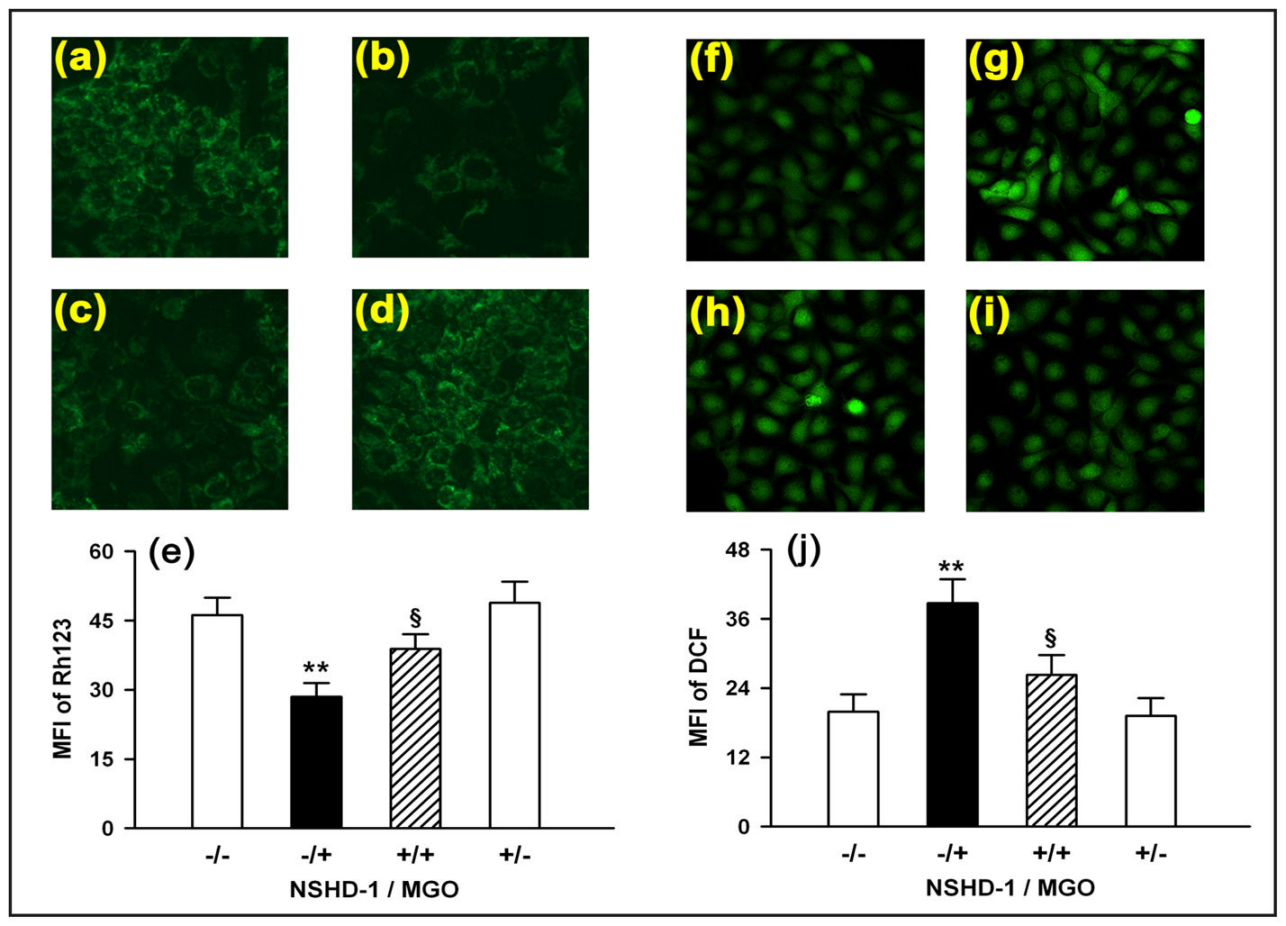
Effects of NSHD-1 on MGO-induced MMP depolarization and ROS accumulation in HaCaT cells Rh123 or DCFH-DA staining followed by photofluorography for the observation of MMP or ROS levels in the cells. (a–d) and (f–i) Random micrographs of Rh123 or DCF-derived fluorescence in the cells of the (a and f) control group, (b and g) treatment with 400 μM MGO for 48 h group, (c and h) treatment with 400 μM MGO for 48 h in the presence of preconditioning with 100 μM NSHD-1 for 1 h group and (d and i) treatment with NSHD-1 alone for 1 h followed by a 48-h culture group. During preconditioning, cell medium of each group contained 300 μM Cys. (e and j) Quantitative analysis of the mean fluorescence intensity (MFI) of Rh123 (group a–d) or DCF (group f–i) using Image J software. Data were shown as the mean ± SE. ^**^P<0.01 versus control group, ^§^*P*<0.05 versus MGO alone group.

Mitochondria play an important role in the activation of apoptotic pathway. MMP depolarization is an early stage of mitochondrial dysfunction, which can be detected by negative MMP-driven entry of the fluorescent dye Rh123. The results of Fig. 8 showed that exposure of HaCaT cells to 400 μM MGO for 48 h obviously suppressed MMP, characterized by a weaker Rh123 fluorescence in the cells of Fig. 8b than that of control group (Fig. 8a). Pretreatment with NSHD-1 alone didn’t alter MMP levels (Fig. 8d), but significantly prevented MGO-induced MMP depolarization (Fig. 8c). This result suggests that the mitochondrial protection of NSHD-1 may contribute to its cytoprotective and antiapoptotic activity. There is a crosstalk between carbonyl stress and oxidative stress since carbonyl agent is able to inhibit mitochondrial oxidative phosphorylation complex. We therefore investigated oxidative status after the indicated treatments. As shown in Fig. 8g, ROS levels were significantly elevated comparing to the control cells (Fig. 8f), and the increased ROS levels were reduced by pretreatment with NSHD-1(Fig. 8h), suggesting its anti-oxidative effects.

### NSHD-1 partially restored cellular adhesion in MGO-treated HaCaT cells

**Fig. 9:**
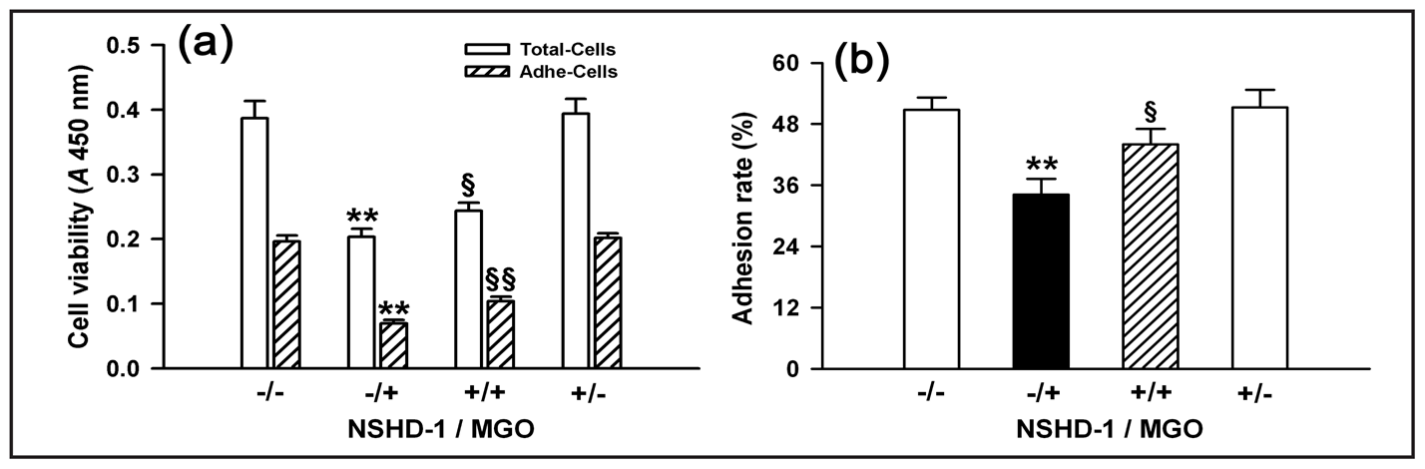
Effects of NSHD-1 on cellular adhesion in MGO-stimulated HaCaT cells The cells were treated with 400 μM MGO for 48 h in the absence or presence of preconditioning with 100 μM NSHD-1 for 1 h. When the cells were preconditioned, the solution of all the groups was prepared in the medium containing 300 μM Cys. After the treatments, the cells were digested with 0.25% trypsin and collected. The harvested cells were inoculated on 96-well plates and cultured. Panel a: CCK-8 assay was performed at the moment of inoculation, and after a further incubation for 12 h to measure the numbers of total cells and adhesive cells, respectively. Panel b: Adhesion rate (%) was calculated by a ratio of adhesion cells to total cells. Data were shown as the mean ± SE. ^**^P<0.01 versus control group, ^§^*P*<0.05, ^§§^*P*<0.01 versus MGO alone group.

Proper keratinocyte adhesion is critical to maintain epidermal integrity and homeostasis. Impaired adhesion is a major reason for chronic and non-healing wound. As shown in Fig. 9a, the treatment with MGO reduced cell viability; however, the adhesive cells had worse viability comparing with that of control group. Furthermore, the result of Fig. 9b displayed that the adhesive ratio (adhesive cells/total cells) in MGO group was lower than that of control group, indicative of a suppressed cellular adhesion. However, prior to the treatment with 400 μM MGO for 48 h, exposure of HaCaT cells to 100 μM NSHD-1 for 1 h significantly prevented the impairment of cellular adhesion.

### NSHD-1 improved cellular migration in MGO-treated HaCaT cells

**Fig. 10:**
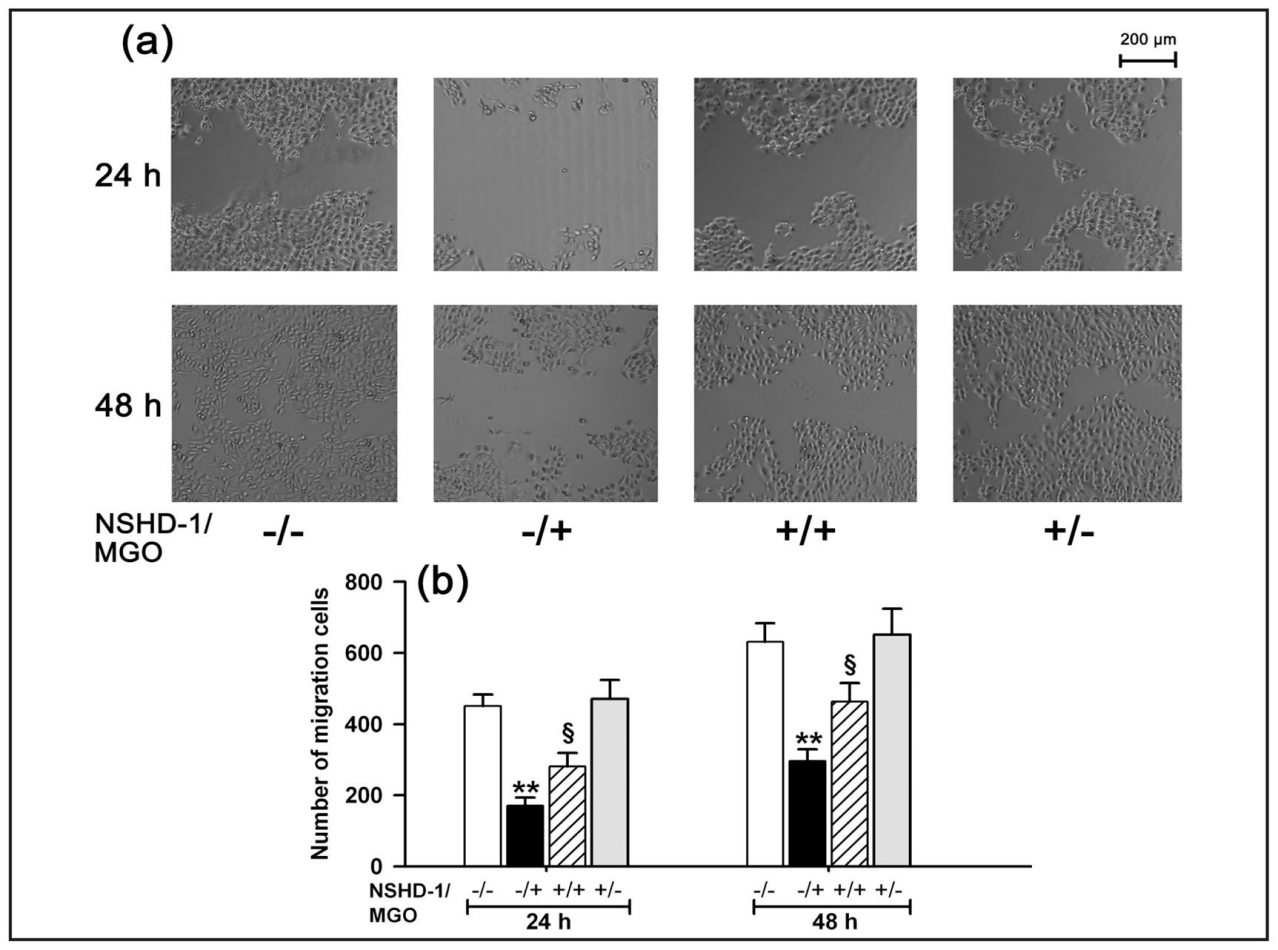
Effects of NSHD-1 on cellular migration in MGO-stimulated HaCaT cells Panel a: The cells were inoculated on 6-well plates and cultured up to 70% confluence. And then they were treated with 400 μM MGO for 48 h in the absence or presence of preconditioning with 100 μM NSHD-1 for 1 h. When the cells were preconditioned, the solution of all the groups was prepared in the medium containing 300 μM Cys. An *in vitro* scratch assay was performed to measure cell migration. The images were captured at 24 h and 48 h after scratch. Panel b: Migration cells was counted in 6 random vision fields of each group using *cell counter module* of Image J software. Data were shown as the mean ± SE. ^**^P<0.01 versus control group, ^§^*P*<0.05 versus MGO alone group.

Keratinocyte migration is conducive to wound repairing. As presented in Fig. 10, under normal condition, the scratch was almost closed within 48 h in the control group. When the cells were treated with 400 μM MGO for 48 h, the scratch was hard to close, indicating an impaired migration. However, before treatment with MGO, the cells were preconditioned with 100 μM NSHD-1 for 1 h and then migration function was examined. The result showed that the impaired migration action was partially restored.

## Discussion

In the present study, it was shown that exposure of human skin keratinocytes, HaCaT cells, to MGO induced cellular injury and behavior dysfunction, as evidenced by decreases in cell viability, MMP, adhesion and migration, as well as increases in apoptotic rate and ROS levels. Furthermore, it was found that the novel controllable donor molecule, NSHD-1, protected HaCaT cells against cellular injury and behavior dysfunction through release of H_2_S.

Evidence has shown that levels of H_2_S are decreased in the plasma of diabetic patients and model rats [14]. Supplementation of H_2_S has emerged as a potential approach to mitigate diabetic injury in high glucose-stimulated endothelial cells [20], pancreatic beta cells [21], and H9c2 cardiomyocytes [22]. In the previous study, we found that added NaHS (a H_2_S donor) protected skin keratinocytes against ischemia-induced injury [15]. Additionally, H_2_S has reportedly enhanced normal mouse wound healing through angiogenic potential in a K_ATP_/MAPK dependent manner [23]. These prompted the exploration of the roles of H_2_S in diabetic skin complications.

Delayed wound healing is a common skin complication of diabetics, which is partially associated with keratinocyte dysfunction. It has been documented that the protection of skin keratinocytes is beneficial to the wound healing in diabetic ulcers [24, 25]. Reportedly, the levels of MGO are elevated in diabetics [7, 8]. In addition, treatment with high concentrations of glucose increased MGO generation in vascular smooth muscle cells [26]. Administration of MGO induced diabetes-like microvascular changes and delayed wound healing [27]. In the current study, MGO was therefore used in conjunction with human skin keratinocytes to create an *in vitro* model of delayed diabetic wound healing. The results indicated that exposure of the cells to MGO at the concentrations of 200 to 600 μM decreased cell viability in a dose-dependent manner confirming the dermal toxicity of MGO. Although the concentration of MGO used in this study is higher than that in the plasma of diabetics, this should still be of scientific interests because the vast majority (approximately 90%) of MGO is bound to macromolecules in tissues [28, 29]. In addition, we noticed that this concentration range was also used in a human skin fibroblast experiment [30]. Importantly, we showed that MGO-induced skin injury was attenuated by preconditioning with NSHD-1. Of note, NSHD-1, as a H_2_S donor, was more stable and controllable, meaning that only in the presence of L-cysteine or reduced glutathion (GSH) this molecule can release H_2_S and that it isn’t easily oxidized by oxygen (O_2_) in atmosphere comparing with NaHS. We believe this is valuable because there are sufficient concentration of L-cysteine [31] and GSH [32] in mammalian cells to activate NSHD-1 and release H_2_S. In addition, this molecule may be superior to GYY4137, another H_2_S donor, since the latter releases H_2_S upon spontaneous hydrolysis, and its ability of H_2_S generation is weak and slow [33].

During wound healing, proper apoptosis is necessary to eliminate some specific cells [34]. However, excessive cellular apoptosis is harmful to the wound healing process. Darby et al. showed that reduced cell proliferation and aberrant control of apoptosis impeded wound healing in genetically diabetic mice [35]. In the present study, we found that treatment of keratinocytes with MGO caused obvious cellular apoptosis, which is in line with the report above [35]. Importantly, MGO-induced apoptotic effects were attenuated by pretreatment with NSHD-1, highlighting the inhibitory effects of H_2_S against apoptosis, which has also been shown in other tissues including brain, lung and heart [36–38]. On the other hand, there are still some studies indicating H_2_S is able to induce apoptosis in smooth muscle cells [39] and insulin-secreting beta cells [40]. We speculate that these distinct effects of H_2_S on apoptosis may be related to tissue specificity.

The mitochondrial pathway plays an important role in apoptosis. A series of experiments were therefore performed to investigate mitochondrial function. Loss of MMP is an early change of mitochondrial dysfunction, and we thus measured MMP using Rh123 staining followed by photofluorography. The results showed that treatment with MGO suppressed MMP levels, while this effect was partially abolished by pretreatment with NSHD-1, suggesting a potent role in mitochondrial protection of H_2_S. Ray and colleagues have demonstrated that MGO inhibits electron flow through complex I leading to mitochondrial dysfunction [41]. In diabetes, it has been believed that prevention of mitochondrial damage may be an important therapeutic strategy [42]. Mitochondrial dysfunction usually causes uncoupling of oxidation and phosphorylation and ROS overproduction. Accumulating evidence has suggested that oxidative status exists in MGO-stimulated cells [29]. It was documented as early as 1993 that ROS levels were markedly increased under the condition of MGO-triggered toxicity in hepatocytes [43]. In this study, we found that exposure of keratinocytes to MGO elicited obvious intracellular ROS accumulation characterized by increased DCF fluorescence. Besides induction of ROS accumulation, MGO can deplete the total glutathione content in rat Schwann cells leading to oxidative imbalance [44]. Through improvement of oxidative imbalance, Vaspin prevented MGO-induced apoptosis in human vascular endothelial cells [45]. However, some study indicated that ROS generation became detectable only under aerobic conditions in severely injured cells, and the author didn’t think ROS were involved in MGO hepatotoxicity [43]. In the following report, the author provided an explanation that these contradictory data may be due to biochemical machinery furnished in various cell types [29]. Interestingly, in this study we also found that the MGO-induced increase in ROS levels was significantly mitigated by pretreatment with NSHD-1, indicating that NSHD-1-triggered cytoprotection may be associated with inhibition of oxidative damage, which is consistent with a previous report [45]. The antioxidation of H_2_S has also been demonstrated in both cellular [46] and *in vivo* models [47] by adding other H_2_S-releasing molecules. According to the previous reports as well as this study, it can be concluded that exposure of human skin keratinocytes to MGO leads to a decrease in viability and an increase in apoptosis, whose mechanisms may be underlain by oxidative stress and mitochondrial impairment. Importantly, NSHD-1 can protect against MGO-induced injury through improvement of mitochondrial function and oxidative status.

Last but not the least, in the light of adhesion and migration involved in delayed diabetic wound healing, we further examined the effects of treatment with MGO on keratinocyte behavior phenotype. The data suggested that exposure of keratinocytes to MGO markedly suppressed cell adhesive function, evidenced by a decreased adhesive rate, which was corroborated by the findings of Tepper et al. on endothelial progenitor cells in type II diabetics [48]. Interestingly, we found that preconditioning with NSHD-1 significantly rescued MGO-induced adhesive impairment in keratinocytes, suggesting NSHD-1 may be beneficial to diabetic wound healing at this point. It has been shown that TGF β, through improvement keratinocyte adhesive action, induced upregulation of extracellular matrix protein betaig-h_3_ contributing to boost wound healing process [49]. Besides adhesive function, cellular migration is also important to wound healing process. This study indicated that exposure of keratinocytes to MGO markedly suppressed cellular migration, which was supported by the decreased keratinocyte mobility observed under hyperglycaemic conditions [24]. A previous study suggested that MGO-induced cellular migration impairment could be associated with oxidative stress [3]. Since the current study suggested that preconditioning with NSHD-1 significantly impeded ROS accumulation in keratinocytes, the effects of NSHD-1 on MGO-induced migration impairment were examined. The results revealed that NSHD-1 in part mitigated the impaired migration induced by MGO in keratinocytes. Notably, very recently, Chen *et al.* found that H_2_S improved wound healing via restoration of endothelial progenitor cell functions and activation of Ang-1 in type 2 diabetic mice [50]. From another point of view, the current study has provided a novel insight into the roles of H_2_S in the improvement of wound healing at the skin keratinocyte level, but contradictory reports on the effect H_2_S on diabetes exist. In streptozotocin-induced diabetic rats, H_2_S signaling pathway was upregulated in the liver and pancreas, which was restored by supplement of insulin [51]. Another *in vitro* study showed that upregulation of the cystathionine gamma-lyase/H_2_S system induced endoplasmic reticulum stress-mediated apoptosis in insulin-secreting beta cells [40]. Therefore, a great many of studies still need to be conducted to bring clarity to the roles of H_2_S in diabetic pathogenesis.

In conclusion, the current study provided novel evidence that a controllable and stable H_2_S donor protects human skin keratinocytes against MGO-related dermal injury and dysfunction. Given the promising findings on the effects of H_2_S, more reasonable H_2_S donors will be synthesized in the future. For instance, active carbonyl group-sensitive and skin permeable H_2_S donor skin sprays could bring relief to patients suffering from delayed wound healing in diabetes mellitus.
